# Estimates of Rel_Seq_, Mesh1, and SAH_Mex_ Hydrolysis of (p)ppGpp and (p)ppApp by Thin Layer Chromatography and NADP/NADH Coupled Assays

**DOI:** 10.3389/fmicb.2020.581271

**Published:** 2020-10-23

**Authors:** Katarzyna Potrykus, Nathan E. Thomas, Bożena Bruhn-Olszewska, Michał Sobala, Maciej Dylewski, Tamara James, Michael Cashel

**Affiliations:** ^1^Department of Bacterial Molecular Genetics, Faculty of Biology, University of Gdańsk, Gdańsk, Poland; ^2^Intramural Program, Eunice Kennedy Shriver National Institute of Child Health and Human Development, National Institutes of Health, Bethesda, MD, United States

**Keywords:** (p)ppGpp, (p)ppApp, RSH, Mesh1, SAH, stringent response, *Methylobacterium extorquens*, *Escherichia coli*

## Abstract

The Mesh1 class of hydrolases found in bacteria, metazoans and humans was discovered as able to cleave an intact pyrophosphate residue esterified on the 3′hydroxyl of (p)ppGpp in a Mn^2+^ dependent reaction. Here, thin layer chromatography (TLC) qualitative evidence is presented indicating the substrate specificity of Mesh1 from *Drosophila melanogaster* and human MESH1 also extends to the (p)ppApp purine analogs. More importantly, we developed real time enzymatic assays, coupling ppNpp hydrolysis to NADH oxidation and pppNpp hydrolysis to NADP^+^ reduction, which facilitate estimation of kinetic constants. Furthermore, by using this assay technique we confirmed TLC observations and also revealed that purified small alarmone hydrolase (SAH_Mex_) from *Methylobacterium extorquens* displays a strong hydrolase activity toward (p)ppApp but only negligible activity toward (p)ppGpp. In contrast, the substrate specificity of the hydrolase present in catalytically active N-terminal domain of the RSH protein from *Streptococcus equisimilis* (Rel_Seq_) includes (p)ppGpp but not (p)ppApp. It is noteworthy that the RSH protein from *M. extorquens* (RSH_Mex_) has been recently shown to synthesize both (p)ppApp and (p)ppGpp.

## Introduction

Bacterial global regulatory stress responses play a major role in their adaptation to constantly changing environmental conditions. One of the best studied responses is the stringent response, characterized by a swift synthesis of large amounts of guanosine tetra- and penta-phosphates, collectively referred to as (p)ppGpp (Potrykus and Cashel, 2008). These second messengers may act through several different modes of action. For example in *Escherichia coli* they directly interact with the RNA polymerase (RNAP) by binding at two distinct sites (Mechold et al., 2013; Ross et al., 2016; Molodtsov et al., 2018), which leads to transcriptional reprogramming allowing for cell survival under harsh conditions. On the other hand, in *Bacillus subtilis* (p)ppGpp accumulation leads to depletion of GTP levels [by using up GTP as a substrate for (p)ppGpp synthesis and by direct inhibition of GTP synthesizing enzymes (Kriel et al., 2012)] that in turn also leads to alterations in gene expression at the transcription initiation level (Krásný and Gourse, 2004; Kriel et al., 2012). Other putative ppGpp targets have been also recently identified in *E. coli* (Wang et al., 2019).

The mode of (p)ppGpp synthesis may differ, depending on the stress condition that triggers its synthesis. For example, two (p)ppGpp synthetases exist in *E. coli* and in other γ- and β-proteobacteria: RelA (active under amino acid deprivation), and SpoT (active under other stresses) (Potrykus and Cashel, 2008). In Firmicutes, as well as in α-, δ- and ε-proteobacteria, (p)ppGpp is synthesized by what we call here RSH proteins (Potrykus and Cashel, 2008) also referred to as Rel (Mittenhuber, 2001; Atkinson et al., 2011), and small synthetases (called SAS, for small alarmone synthetase) seemingly devoid of large regulatory domains (Lemos et al., 2007; Nanamiya et al., 2008; Atkinson et al., 2011; Steinchen et al., 2018).

Still, no matter what the mode of (p)ppGpp synthesis is, regulation of (p)ppGpp hydrolysis is equally crucial for the cell, so that it can quickly respond once the environmental conditions improve. In *E. coli* (and other γ- and β- proteobacteria) that function is carried out by SpoT, while in Firmicutes, α-, δ- and ε-proteobacteria it is carried out by long bifunctional RSH enzymes. Interestingly, in the latter case, stand-alone (p)ppGpp hydrolases called SAH (for small alarmone hydrolases) have been also identified by bioinformatics methods (Atkinson et al., 2011). These enzymes are related to Mesh1 enzymes found in the metazoan species, such as *Drosophila melanogaster*, worms, mice, and humans (Sun et al., 2010). There, it was demonstrated that the *D. melanogaster* Mesh1 and human MESH1 enzymes (for Metazoan SpoT Homolog-1) are structurally similar to the hydrolysis domain of an RSH enzyme from *Streptococcus equisimilis* (Rel_Seq_) and are capable of ppGpp hydrolysis, although no source of ppGpp synthesis in metazoa has been discovered. The authors had noted that the Mesh1 enzyme requires Mn^2+^ and reverses the toxicity of RelA induction due to ppGpp synthesis when expressed in *E. coli*, as well as in *D. melanogaster* tissue culture cells (Sun et al., 2010). Yet, bacterial SAH enzymes have been neglected for several years, with only one recent report characterizing SAH from *Corynebacterium glutamicum* (Ruwe et al., 2018).

In this study, we report findings accumulated over the past several years in the Cashel and the Potrykus labs. Our interest arose from several leads. First, as mentioned above, (p)ppGpp hydrolysis regulation is as important as its synthesis. For many years the Cashel lab has been investigating the SpoT and Rel_Seq_ hydrolysis regulation (e.g., Gentry and Cashel, 1995; Mechold et al., 2002). Second, the discovery of Mesh1 with concomitant inability to demonstrate (p)ppGpp synthesis in metazoa, has led both labs to search for a different substrate for these enzymes. An obvious candidate was ppApp, a structural analog of ppGpp. This nucleotide has been observed long ago in *B. subtilis*, and its synthesis was thought to be carried out by factors associated with ribosomes under starvation conditions inducing sporulation (Rhaese et al., 1977; Nishino et al., 1979). Later, the role of (p)ppGpp in sporulation was shown as due to indirect effects on GTP pool depletion (Ochi et al., 1982), while the (p)ppApp occurrence was deemed as an experimental artifact, and thus this nucleotide has been neglected for many years.

As we discuss later, we confirm that Mesh1 and MESH1 hydrolyze ppGpp (as reported by Sun et al. (2010), but also find that pppGpp and (p)ppApp can serve as substrates. These findings have led the Potrykus lab to wonder if the same would be true for bacterial SAH enzymes, and whether (p)ppApp might serve as a second messenger, in parallel to (p)ppGpp. *Methylobacterium extorquens* (a SAH- bearing bacterium) and *E. coli* have been chosen as the model organisms. As we reported elsewhere, we found pppApp to be synthesized by *M. extorquens* and *E. coli* cells *in vivo*, and RSH_Mex_ enzyme (a long bifunctional RSH protein) is the source of both, (p)ppGpp and pppApp in *M. extorquens* (Sobala et al., 2019). At the same time, we confirmed and dissected the role of (p)ppApp in regulating *E. coli* RNAP activity at the ribosomal *rrnB* P1 promoter (Bruhn-Olszewska et al., 2018).

Here, we report our initial findings on the Mesh1 and MESH1 degradation of pppGpp and (p)ppApp, supplemented by later findings for the SAH enzyme from *M. extorquens* AM1 strain (SAH_Mex_). Interestingly, the *M. extorquens* SAH enzyme is active toward (p)ppApp but not (p)ppGpp. Moreover, our joint investigation has led the Cashel lab to develop a real time kinetic optical assay by coupling NADH oxidation or NADP+ reduction to monitor hydrolysis of nucleotide tetraphosphates or pentaphosphates, respectively. Compared to thin layer chromatography (TLC) or HPLC methods where the reaction needs to be terminated to make a reading, these coupled assays generate real time data that greatly facilitates estimating kinetic constants for (p)ppGpp and (p)ppApp hydrolysis. Here, this method is used to document all three possible classes of hydrolase substrate specificities: Rel_Seq_ is active toward (p)ppGpp but not (p)ppApp; SAH_Mex_ is active toward (p)ppApp but not (p)ppGpp; and the Mesh1 enzyme from *D. melanogaster* hydrolyzes both.

## Materials and Methods

### Strains and Plasmids

Plasmid pUM77, a pET21 derivative, was used as source of Rel_Seq__1__–__385_ (catalytically active N-terminal fragment containing a C-terminal his-tag) (Mechold et al., 2002). Mesh1 (*D. melanogaster*) and MESH1 (human) were overexpressed from pET28 derivatives (Sun et al., 2010). For SAH_Mex_ overexpression, sequences of the SAH encoding gene from *M. extorquens* AM1 strain (GenBank locus tag: MexAM1_META1p3226) was optimized for GC content and codon usage for *E. coli* (GeneArt Strings service, Thermo Scientific) the synthetic DNA fragment was cloned into pCIOX (pET28 derivative; a gift from Dr. Andrea Mattevi, Addgene plasmid #51300), yielding plasmid pKP2117.

In all cases, BL21 (λDE3) was used for protein overexpression. *Streptomyces morookaensis* (ATCC#19166) was used as the source of the promiscuous pyrophosphotransferase capable of the βγ-pyrophosphate transfer from ATP or GTP onto the ribosyl-3′ hydroxyl group of any purine nucleotide (Oki et al., 1975). Plasmid and strain requests should be sent to K. Potrykus.

### Protein Purification

Rel_Seq_1-385 was purified as described in Mechold et al. (2002). Mesh1 and MESH1 were purified according to Sun et al. (2010), with slight modifications. Briefly, BL21(λDE3) cells transformed with appropriate pET28a-derived plasmids, were grown in LB and when the culture reached OD_600_∼0.5, protein overproduction was induced with 1 mM IPTG for 1 h. The cells were spun, resuspended in the lysis buffer (20 mM β-ME, 50 mM NaPO_4_ pH 8.0, 0.5 M NaCl, 10% glycerol, 20 mM imidazole), supplemented by lysozyme and a tablet of Complete Protease Inhibitor Cocktail (Roche), incubated on ice for 30 min, and then disrupted by sonication. After clearing by centrifugation, the supernatants were batch-adsorbed onto Ni^2+^-NTA agarose (Qiagen) or TALON-agarose (Clontech). Next, the resins were washed with the wash buffer (the same as lysis buffer, but containing 40 mM imidazole), and the proteins were eluted with the same buffer but containing 300 mM imidazole. All fractions were checked by SDS-PAGE, appropriate fractions were pooled and dialyzed against thrombin-cleavage buffer (20 mM Tris–Cl pH 8.0, 150 mM NaCl, 5 mM CaCl_2_, 5% glycerol, 0.5 mM β-ME). His-tags were removed with the Thrombin Capture Kit, Novagen. The fractions were then dialyzed against 2× storage buffer (100 mM Tris–Cl pH 8.0, 500 mM NaCl), and then glycerol and DTT were added to 50% and 2 mM, respectively.

SAH_Mex_ was purified under similar conditions, except that the His8-SUMO tags were cleaved with in-house purified his-tagged-Ulp1 SUMO protease. To this end, after initial fractionation and purification, the pooled fractions were dialyzed against the following buffer: 20 mM Tris–HCl pH 8.0, 250 mM NaCl, 10% glycerol, and processed as described for the His8-SUMO tagged proteins in Sobala et al. (2019). The storage buffer was the same as for the Mesh1 and MESH1 proteins mentioned above.

### (p)ppNpp Preparation

The (p)ppNpp standards were prepared and purified generally as described in Bruhn-Olszewska et al. (2018). In detail, the *S. morookaensis* extract was prepared by inoculating 500 ml of LB in flasks containing glass beads (∼2–3 mm in diameter) to disperse the culture. After overnight cultivation, the cultures were centrifuged, and Tris–Cl (pH 8.0) was added to the collected supernatants to 50 mM. Next, the pyrophosphotransferase fraction was precipitated with ammonium sulfate in the cold room (200 g NH_2_SO_4_/500 ml extract) with stirring for 30 min. After centrifugation, the pellet was resuspended in 11 ml of 10 mM Tris–Cl pH 8.0, 0.1 mM EDTA, 10% glycerol, followed by overnight dialysis against 800 ml of the same buffer, in the cold, using Slide-a-lyzer Cassettes (Pierce, cut-off 10 kDa). After dialysis, the extract can be either used directly or stored at 4°C (stable for at least 6 months).

The (p)ppNpp synthesis reactions were prepared in the following buffer: 50 mM Tris–Cl pH 8.0, 0.5 mM EDTA, 20 mM MgCl_2_, 9 mM donor NTP (ATP), and 9 mM acceptor (ATP, ADP, GTP or GDP). Depending on the *S. morookaensis* extract activity, the extract was added to 1/8–1/2 of the final volume. Reactions were carried out at 30–37°C for 15–60 min, and stopped by adding 1/4 volume of phenol and 1/10 volume of chloroform. After vortexing and centrifugation, the supernatant was collected and LiCl was added to 2 M. Nucleotides were precipitated by adding five volumes of 96% EtOH, stored overnight at −20°C, and centrifuged. The pellets were air-dried, resuspended in 25 mM Tris–Cl pH 8.0, 0.5 mM EDTA, 0.1 M LiCl, and applied on a QAE A25 Sephadex column. The column was washed first with the same buffer, and then nucleotide fractions were eluted with a linear gradient of 0.1–0.5 M LiCl in the same buffer. Each fraction was monitored by UV_260_ and checked for purity by TLC. The final purified fractions were pooled, adjusted to 3 M LiCl, and 5 volumes of 96% EtOH were added, followed by aliquoting, precipitation at −20°C and centrifugation. The resulting nucleotide pellets were washed at least twice with 80% EtOH, air dried and stored at −20°C until use (stable for at least a year). To use, pellets were resuspended in 25 mM Tris–Cl pH 8.0, 0.05 mM EDTA. Nucleotide concentrations are estimated by absorbance at 260 nm for (p)ppApp (ε = 15 mM^–1^ cm^–1^), and at 254 nm for (p)ppGpp (ε = 13.7 mM^–1^ cm^–1^).

### (p)ppNpp Hydrolysis Tests by Thin Layer Chromatography (TLC)

These reactions were carried out in the following buffer: 5 mM (p)ppNpp, 50 mM Tris–Cl (pH 8.0), 250 mM NaCl, 14 mM MgCl_2_ and 6.5 mM MnCl_2_. Mesh1, MESH1 and SAH_Mex_ were added to 0.025 μg/μl, and Rel_Seq_1-385 was used at 1 μg/μl (final concentration). The reactions were carried out at 37°C and were stopped at indicated times by addition of an equal volume of 2 M formic acid. TLC was performed by spotting samples on PEI- cellulose plates (Merck). To resolve the samples, 1.5 M KH_2_PO_4_ buffer (pH 3.4) was used. The plates were viewed under UV_254_ light.

### NADP^+^/NADH Coupled Assay

For the ppApp and ppGpp hydrolysis assays, the reactions contained: 50 mM Tris–HCl (pH 8.0), 200 mM NaCl, 50 mM KCl, 5 mM MgCl_2_, 1 mM MnCl_2_, 60 μM EDTA, 300 μM NADH, 5 mM phosphoenolpyruvate (PEP), 6 U/ml pyruvate kinase (PK), and 6 U/ml lactate dehydrogenase (LDH). For pppGpp assays, the reactions contained: 50 mM Tris–HCl (pH 8.0), 200 mM NaCl, 50 mM KCl, 5 mM MgCl2, 1 mM MnCl_2_, 60 μM EDTA, 300 μM NADP^+^, 1.1 mM glucose, 5 U/ml hexokinase (HK) and 4 U/ml G6P dehydrogenase (G6PD). In case of pppApp hydrolysis, the reaction buffer was the same as for pppGpp, except that 500 μM ADP and 15 U/ml nucleoside diphosphate kinase (NDK) were also added. All of the above reagents and enzymes were purchased from Sigma-Aldrich. The initial reaction volume was 180 μl and the concentrations given above were calculated for that volume. In all cases, Mesh1 and SAH_Mex_ were added to 75 ng/reaction (18 nM), and Rel_Seq__1__–__385_ was used at 1.6 μg/reaction (0.176 μM).

The reaction work-up was as follows: 180 μl of the reaction buffers, already containing enzymes to be tested for hydrolysis, were aliquoted into 96 well-plates and pre-warmed to 37°C for 15 min. Next, 20 μl of (p)ppNpp solutions at appropriate (10×) concentrations were dispensed, so that the final tested concentrations were: 0, 62.5 μM, 125 μM, 250 μM, 500 μM, and 1 mM. Reaction progress was monitored by measuring absorbance at 352 nm, an approximation of the ideal 340 nM necessitated by available filters. For a 200 μl well, the extinction coefficient of NAD(P)H is 3100 M^–1^. Readings were taken automatically at close intervals (every 15–30 s) for 30 to 40 min. Synergy HT (BioTek) or EnSpire (Perkin Elmer) plate readers were used. All reactions were always carried out at least in triplicate.

### NADPH Hydrolysis Tests

In these assays, scheme for (p)ppNpp hydrolysis was followed in order to produce NADPH, except that ATP was added directly to the assay and (p)ppNpp’s were omitted. All enzyme concentrations (if employed) and buffer conditions were the same as for the standard coupled enzymatic assay except where noted otherwise. First, glucose-6-P was produced by using HK and glucose in the presence of 0.5 mM ATP. The reaction was incubated at 37°C for 20 min. Full conversion of ATP into ADP was monitored by TLC. Next, NADP^+^ (0.5 mM, final concentration) was added to initiate NADPH production by G6PDH. The reaction was carried out at 37°C for 20 min, and full conversion of NADP+ into NADPH was also monitored by TLC. Then, Mesh1, MESH1 or SAH_Mex_ were added at 4 ng/μl (0.2 μM final) and the reactions were allowed to proceed for 30 min at 37°C, and then stopped by addition of formic acid to 1 M. Samples were spotted on PEI-cellulose TLC plates and resolved in a LiCl step gradient (0.2 M LiCl for the first 2 cm; 1 M LiCl for the next 4 cm; 1.6 M LiCl for the final 6.5 cm).

For the malachite green assay, the same conditions were used, except that the tested proteins were used either at 18 nM or 0.2 μM concentrations, and ATP and NADP+ were added to 0.3 mM. Samples were withdrawn at 5, 10, and 30 min after Mesh1, MESH1 or SAH_Mex_ addition, and the reactions were stopped by adding formic acid to 1 M. 20 μl of each reaction were diluted to 400 μl with water, and then 100 μl of the malachite green reagent were added, freshly prepared according to Baykov et al. (1988); the assay was carried out as described in that report. Standard curve was prepared in the same reaction buffer as the assays, and known concentrations of NaH_2_PO_4_ were used. Under these conditions, detection limit was established as 5 μM free phosphate.

### Data Analysis

Initial reaction rates for the kinetic assays were determined by linear regression with the use of Microsoft Excel software. Kinetic constants were estimated with the KaleidaGraph software by plotting initial reaction rates against substrate concentration, and using non-linear regression to fit the Michaelis-Menten equation. Prior to plotting in KaleidaGraph, the initial reaction rate data were corrected for the expected basal assay activity. This basal activity was established by estimating NADH oxidation or NADP+ reduction in the absence of the (p)ppNpp hydrolase, but in the presence of a given pppNpp or ppNpp, respectively (discussed below and Supplementary Figure S1).

## Results and Discussion

### Qualitative Estimates of (p)ppNpp Hydrolysis by Thin Layer Chromatography

**FIGURE 1:**
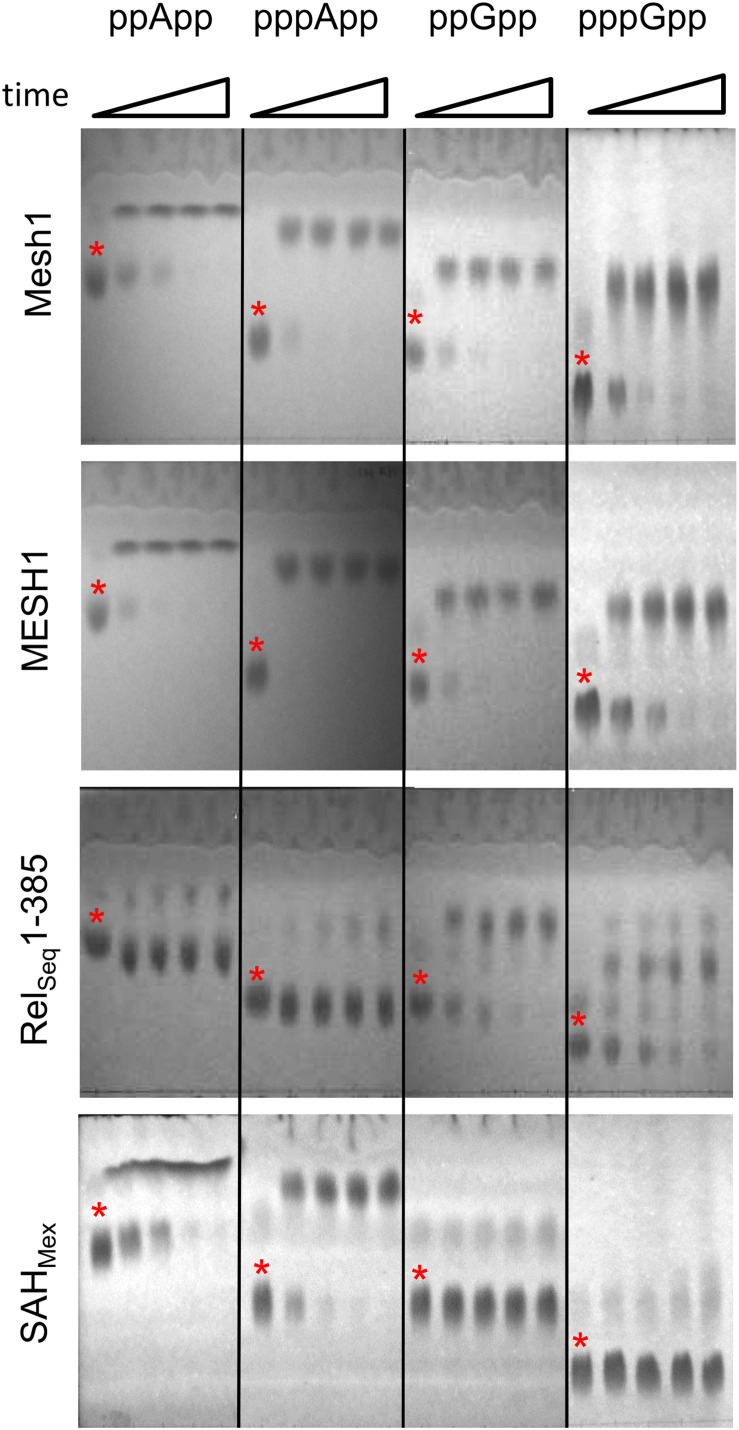
Thin layer chromatography analysis of hydrolytic activities of Mesh1 (*D. melanogaster*), MESH1 (*Homo sapiens*), Rel_Seq_1-385, and SAH_Mex_. Hydrolysis of (p)ppApp and (p)ppGpp was monitored over time (0, 5, 10, 20, and 30 min). Samples were spotted on PEI-cellulose plates, resolved in the 1.5 M KH_2_PO_4_ buffer (pH 3.4) and visualized under UV light. Asterisks are placed just above spots corresponding to the given (p)ppNpp time 0 controls. Products of ppApp, pppApp, ppGpp, and pppGpp hydrolysis are ADP, ATP, GDP, and GTP, respectively.

Figure 1 documents TLC assays of (p)ppApp and (p)ppGpp hydrolysis by the *Drosophila* Mesh1 protein, human MESH1, SAH_Mex_ and Rel_Seq_1-385. Standard development conditions for PEI cellulose TLC were used with 1.5 M KH_2_PO_4_ (pH 3.4) buffer, followed by visualization under UV_254_ light. The chromatograms indicate that Mesh1 is able to hydrolyze all four substrates tested, i.e., ppApp, pppApp, ppGpp, and pppGpp. It seems that pppApp is the most efficiently hydrolyzed substrate. The human MESH1 hydrolase displays similar activities as shown for *Drosophila* Mesh1. Surprisingly, the SAH_Mex_ protein is able to hydrolyze (p)ppApp but not (p)ppGpp. For SAH_Mex_, pppApp seems to be hydrolyzed faster than ppApp, as was observed for Mesh1. The Rel_Seq_1-385 protein hydrolytic activity toward guanosine derivatives is confirmed as expected (Mechold et al., 2002), but it is exceptional in that this enzyme displays only negligible activity toward (p)ppApp. Quantitative activity comparisons of the different enzymes require determining their kinetic constants.

It is noteworthy that the standard TLC resolution of (p)ppGpp and (p)ppApp using PEI cellulose and 1.5 M KPi (pH 3.4) is found to be inadequate for clearly distinguishing between co-migrating pairs: ppApp and GTP; pppApp and ppGpp; and possibly pApp and GDP (Sobala et al., 2019). A more rigorous comparison comes from using 2-dimensional TLC (Sobala et al., 2019), however, when using known substrates, a 1-D TLC is still sufficient.

Similarly, currently, a rigorous analysis of complex nucleotide mixtures in cell extracts appears to require HPLC coupled to mass spectrometry or recently reported capillary electrophoresis, capable of detecting all four (p)ppNpp’s in one run (Haas et al., 2020). Still, determining kinetic constants with these methods would be very time consuming, as each reaction for a given nucleotide concentration would have to be stopped at several time points, and then each would have to be processed separately. Thus, for purified enzymes and known substrates, a coupled enzymatic assay is more useful, where the reaction rate can be followed in real-time.

### Coupled Enzymatic Assay Rationale

**FIGURE 2:**
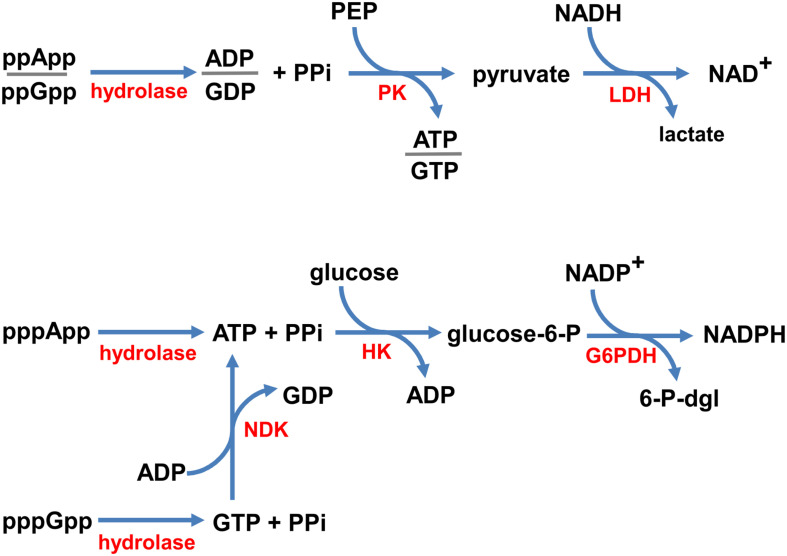
Coupled enzymatic reaction schemes. ppNpp hydrolysis is coupled to the disappearance of NADH (top), and pppNpp hydrolysis is coupled to the appearance of NADPH (bottom), monitored in real time as changes in absorbance at 340 nm. Enzymes are denoted in red (hydrolase – the investigated enzyme; PK, pyruvate kinase; LDH, lactate dehydrogenase; NDK, nucleotide kinase; HK, hexokinase; G6PDH, glucose-6-phosphate dehydrogenase) and substrates/products are in black (PPi, pyrophosphate; PEP, phosphoenolpyruvate; glucose-6-P, glucose-6-phosphate; 6-P-dgl, 6-phospho-D-glucono-1,5-lactone).

In order to devise a real-time enzymatic assay to monitor (p)ppNpp hydrolysis, we adapted classical NADH oxidation and NADP+ reduction assays for near UV optical measurements. Schemes illustrating the reaction pathways of our enzyme coupled assays are shown in Figure 2.

Since, the products of ppGpp and ppApp hydrolysis are GDP and ADP, respectively, we coupled these enzymatic reactions with the NADH oxidation assay. In our set-up, NADH is oxidized to NAD^+^ by LDH *via* production of pyruvate by PK from PEP and ADP or GDP. This approach was possible because of the broad specificity of PK, which phosphorylates GDP about 70% as efficiently as ADP (Plowman and Krall, 1965). Thus, the same set of buffer and enzyme conditions can be used to follow both, the ppApp and ppGpp hydrolysis.

On the other hand, products of pppApp and pppGpp hydrolysis are ATP and GTP, respectively. Accordingly, we coupled hydrolysis of pppApp to NADP+ reduction by glucose-6-phosphate dehydrogenase (G6PD) *via* production of glucose-6-phosphate (G6P) by hexokinase (HK) from ATP and glucose. Unlike PK, HK is very specific for ATP, and GTP cannot serve as a substrate. Thus, to follow pppGpp hydrolysis, it was necessary to add nucleoside diphosphate kinase (NDK) and ADP, which catalyzes the reactions of forming ATP and GDP from ADP and GTP, respectively. Since ATP is then recycled back into ADP by the hexokinase reaction, it is only necessary to add catalytic amounts of ADP.

In summary, ppNpp hydrolysis ultimately leads to depletion in NADH levels, which we monitored by measuring a drop in absorbance at UV_352_, while pppNpp hydrolysis is followed by monitoring accumulation of NADPH, which is monitored by an increase in absorbance at the same wavelength. Examples of raw data obtained with these assays are shown in Supplementary Figure S2.

### Coupled Enzymatic Assay Reaction Controls

Several potential side reactions could invalidate the coupled reactions shown in Figure 2. One class of errors arises from structural similarities between ppApp and ADP, and another from similarities between pppApp and ATP. For example, PK might use ppApp instead of ADP, or ppGpp instead of GDP. A similar scenario is that pppApp or pppGpp might substitute for ATP in the hexokinase catalyzed reaction. Either of these side reactions could break the coupled assay, if substantial. If modest, the extent of the reaction could be used as a correction factor.

Control reactions for ppNpp hydrolysis assay led to the finding that indeed PK may remove phosphate group from PEP to phosphorylate either ppApp to pppApp or ppGpp to pppGpp, yielding pyruvate that can be used in the downstream reaction. Calculated Vmax values are almost identical for both ppNpp’s: 0.38 ± 0.06 μM/min for ppApp, and 0.38 ± 0.01 μM/min for ppGpp (Supplementary Figure S1).

In case of the pppNpp hydrolysis assay, we found that HK can catalyze a transfer of a phosphate to glucose from pppApp with a very slow rate (Vmax = 0.066 ± 0.009 μM/min; Supplementary Figure S1). Similarly, pppGpp may be used by either HK or NDK as a phosphate group donor to yield G6P or ATP, respectively, although this is also a slow reaction (Vmax = 0.075 ± 0.008 μM/min; Supplementary Figure S1). Correction for this side reaction involves subtracting individual data points obtained without added hydrolase from data with hydrolase and recalculating kinetic constants.

An additional control is needed to be sure that the capacity of the coupled assay component concentrations is not exceeded by excessive hydrolytic activity of added enzymes. To achieve this, small titrated amounts (50–60 μM) of immediate hydrolysis products, ADP, GDP, or ATP, were added to the coupled reactions but without hydrolase and the activities were measured. If the total assay capacity is not exceeded then coupled activities with even the small levels of these products should sustain substantially greater activities than those measured with hydrolase at higher substrate levels. Supplementary Figure S3 indicates initial rates for ATP (36 μM/min), ADP (81 μM/min), and GDP (60 μM/min); as demonstrated in the following sections, rates achieved with the tested (p)ppNpp hydrolases were much slower (Vmax values ranged from 1.1 to 26 μM/min for hydrolysis of (p)ppApp and (p)ppGpp by all enzymes). This means that the activities of enzymes used in the coupled assay itself are not limiting to determine accurate rates of (p)ppNpp hydrolysis by the enzymes investigated here.

### Mesh1 Hydrolyzes ppApp and pppApp Equally Well, While ppGpp Is the Least Efficiently Used Substrate

**FIGURE 3:**
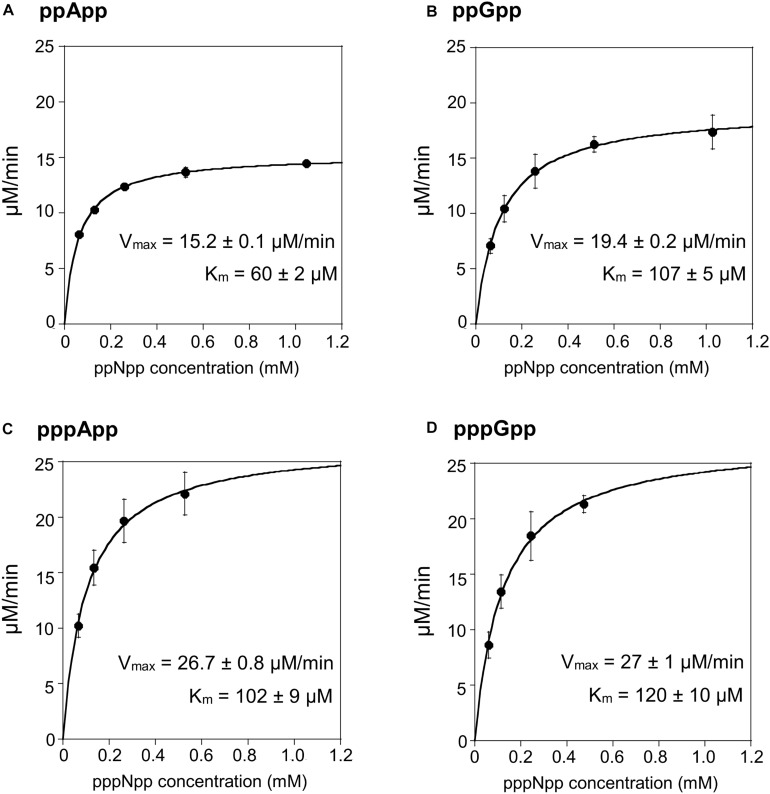
(p)ppApp and (p)ppGpp hydrolysis by Mesh1. Non-linear regression of rate vs. (p)ppNpp concentration. **(A)** ppApp, **(B)** ppGpp, **(C)** pppApp, and **(D)** pppGpp. The kinetic constant calculations are corrected for hydrolysis-independent activity.

In order to test the enzymatic coupled assay, we first decided to employ the *Drosophila* Mesh-1 enzyme which in our initial hydrolysis tests visualized by TLC was shown to be active toward both, (p)ppGpp and (p)ppApp. Examples of raw data obtained for this enzyme are shown in Supplementary Figure S2, while the processed data is presented in Figure 3 and Table 1.

**TABLE 1:** Summary of kinetic constants for (p)ppNpp hydrolases, estimated by the coupled enzymatic assay.

**Enzyme**	**Substrate**	**Vmax (μM/min)**	**Km (μM)**	**kcat (s^–1^)**	**kcat/Km (s^–1^M^–1^)**
Mesh1	ppApp	15.20.1	602	13.60.01	2.3 ± 0.1 × 10^5^
	pppApp	26.70.8	1029	23.90.64	2.4 ± 0.2 × 10^5^
	ppGpp	19.40.2	1075	17.40.17	1.6 ± 0.1 × 10^5^
	pppGpp	27.01	12010	24.00.89	2.0 ± 0.2 × 10^5^
Rel_Seq_ 1-385	ppApp	*no activity*	*no activity*	*no activity*	*no activity*
	ppGpp	1.70.1	6010	0.161.7	3.0 ± 1.0 × 10^3^
SAH_Mex_	ppApp	1.30.1	11426	1.190.1	1.05 ± 0.1 × 10^4^
	pppApp	6.10.4	22237	5.490.3	2.47 ± 0.2 × 10^4^
	ppGpp	*no activity*	*no activity*	*no activity*	*no activity*
	pppGpp	*no activity*	*no activity*	*no activity*	*no activity*

**FIGURE 4:**
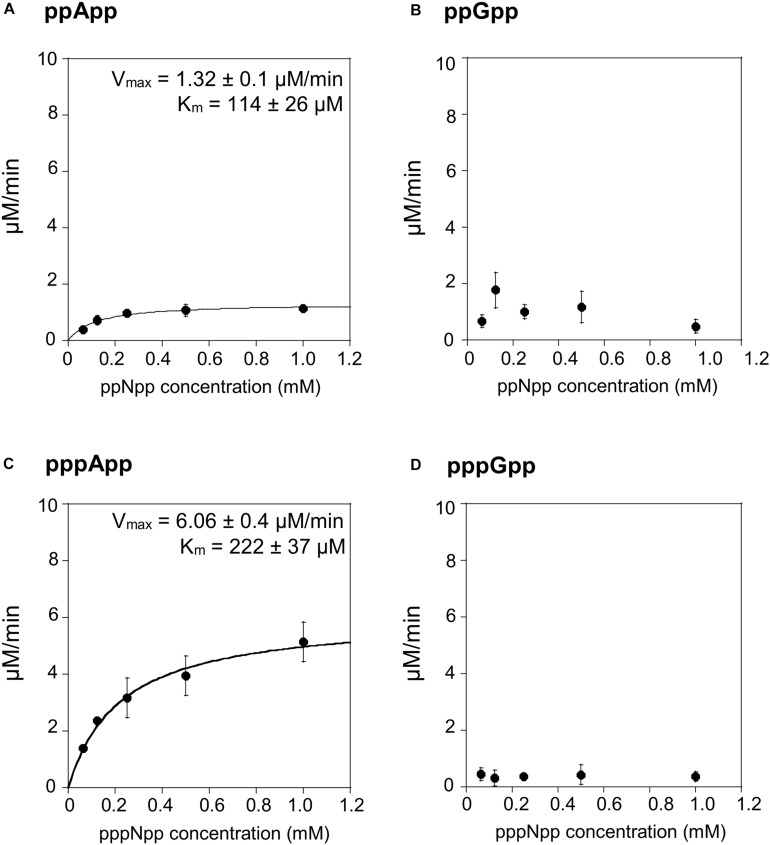
(p)ppApp and (p)ppGpp hydrolysis by SAH_Mex_. Non-linear regression of rate vs. (p)ppNpp concentration. **(A)** ppApp, **(B)** ppGpp, **(C)** pppApp, and **(D)** pppGpp. The kinetic constant calculations are corrected for hydrolysis-independent activity.

**FIGURE 5:**
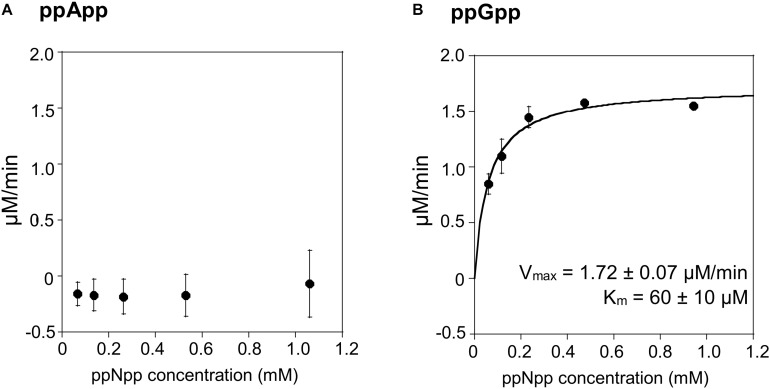
ppApp and ppGpp hydrolysis by Rel_Seq_. Non-linear regression of rate vs. (p)ppNpp concentration. **(A)** ppApp, **(B)** ppGpp. The kinetic constant calculations are corrected for hydrolysis-independent activity.

*Calculations for Mesh1, Rel_*Seq*_1-385, and SAH_*Mex*_ are based on data presented in Figures 3–5. Error estimates are rounded off from values presented in these figures. V_*max*_ and K_*m*_ were calculated using the KaleidaGraph software.*

The Mesh1 hydrolysis rates measured for both ppApp and ppGpp fit well to Michaelis-Menten kinetics over the entire range of substrate concentrations tested. This was also true for pppApp and pppGpp but only for substrate concentrations ranging from approximately 50 to 500 μM. The highest substrate concentration (1000 μM) gave consistently lower rates in the pppNpp assay. Therefore, in Figure 3 the data from the highest pppNpp substrate concentration was excluded and this value is not used for calculating V_max_ and K_m_. The reason for this anomaly is unclear.

The comparative results shown in Table 1 indicate that V_max_ and K_m_ values for Mesh-1 hydrolysis for both adenosine or guanosine pentaphosphate derivatives are not very different (26.7 vs. 27.0 μM/min [V_max_], and 102 vs. 120 μM [K_m_]). For the tetraphosphate substrates, the V_max_ for ppGpp (19.4 μM/min) is 1.27-fold higher than for ppApp (15.2 μM/min), while the Michaelis constant for ppApp (60 μM) is 1.78-fold lower than for ppGpp (107 μM). Still, k_cat_ seems to compensate for these differences, and when comparing the overall catalytic efficiency of the enzyme (k_cat_/K_m_ [s^–1^M^–1^]) it is evident, that the enzyme is hydrolyzing ppApp and pppApp equally well, while ppGpp is the least efficiently used substrate.

### SAH_Mex_ Hydrolyzes pppApp More Efficiently Than ppApp, and Displays Only Negligible Activity Toward (p)ppGpp

We then tested the hydrolysis activity of SAH_Mex_, which based on the TLC assays (Figure 1) was expected to hydrolyze (p)ppApp nucleotides but not (p)ppGpp. Indeed, these results were confirmed with the coupled enzymatic assay (Figure 4 and Table 1). Also, in both cases, SAH_Mex_ is more efficient at hydrolyzing pppApp than ppApp, even though this enzyme’s Km for pppApp is 1.9-fold higher than for ppApp (222 vs. 122 μM). Apparently, the enzyme compensates for this by having a 4.6-fold higher V_max_ in case of pppApp than ppApp (6.1 vs. 1.3 μM/min), which ultimately leads to 3.7 higher catalytic efficiency toward the pentaphosphate derivative than the tetraphosphate when calculating kcat/Km (2.47 ± 0.2 × 10^4^ s^–1^M^–1^ vs. 1.05 ± 0.1 × 10^4^ s^–1^M^–1^).

The fact that SAH_Mex_ is more efficient at hydrolyzing pppApp than ppApp is very interesting in the light of a recent discovery that the RSH enzyme from *M. extorquens* (RSH_Mex_) synthesizes pppApp but not ppApp *in vitro*; this was also true for *in vivo* assessment of *M. extorquens* produced (p)ppNpps–only pppApp, ppGpp, and pppGpp were detected (Sobala et al., 2019). This highlights the complexity of the evolved systems regulating (p)ppNpp synthesis and degradation. However, it cannot be excluded that under certain conditions, ppApp might be still produced.

### (p)ppNpp Hydrolysis by Rel_Seq_1-385

The TLC assays presented in Figure 1, led to the expectation that the coupled ppGpp hydrolysis activities displayed by the Rel_Seq_1-385 hydrolase would be higher than for ppApp because the ADP hydrolysis product was barely evident even after prolonged incubation. The results of the enzymatic coupled assay shown in Figure 5 quantitatively validate the TLC assay. After correction for the basal assay activity there is no evident hydrolysis activity toward ppApp while appreciable ppGpp hydrolysis persists. We estimate that this enzyme’s kinetic constants are as follows: Km for ppGpp is 60 ± 10 μM, while V_max_ is 1.7 μM/min. The overall Rel_Seq_1-385 fragment hydrolytic activity is rather low when calculating kcat/Km (3 ± 1 × 10^3^ s^–1^M^–1^), although this is not entirely unexpected since it was reported previously that this protein has a lower hydrolase activity than full-length RelSeq (Mechold et al., 2002).

It is intriguing that the tested enzymes display such diverse activities toward (p)ppNpp’s. Supplementary Figure S4 shows amino acid sequence comparison between these enzymes. It is not evident which residues might be responsible for the base specificity (A, G or both). However, in close proximity to the key conserved residues, there are several examples of residues that are not present in enzymes unable to hydrolyze (p)ppApp and those unable to hydrolyze (p)ppGpp. Taking RelSeq sequence as the reference, examples of the former are: T36A, V54N, C77A, R149K, and M153L; and of the latter: I57A, V84L, D80Q, and L150T. It may be that they affect orientation of the key residues and thus affect the enzyme’s specificity. On the other hand, another set of possible candidates concerns those residues that are only present in Mesh1 and MESH1 (able to hydrolyze both, (p)ppApp and (p)ppGpp) but differ in SAH_Mex_ and Rel_Seq_. Clearly, further investigations are needed to resolve this problem.

### NADPH Does Not Seem to Be Hydrolyzed by Mesh1, MESH1, or SAH_Mex_ Under Coupled Assay Conditions

Recently, is has been suggested that that the human MESH1 enzyme is an NADPH phosphatase that degrades NADPH to NADH (Ding et al., 2020). Since in the coupled assay for pppNpp hydrolysis NADPH accumulation is being followed (see Figure 2), this could have a potential impact on the obtained results. Even though we would not observe a change in A_352_ because NADH and NADPH display the same absorption profile, there could be an impact on the kinetic values observed due to substrate competition between NADPH and pppNpp for Meshl/SAH binding.

**FIGURE 6:**
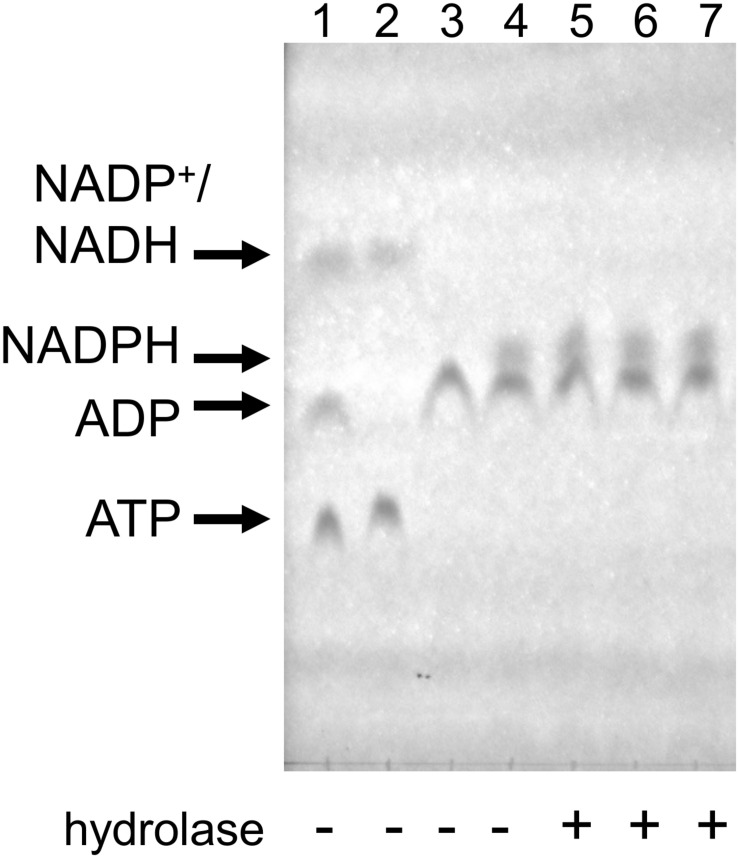
NADPH does not seem to be hydrolyzed by Mesh1, MESH1, and SAH_Mex_ under the coupled assay conditions when visualized by TLC assay. Reactions were carried under conditions described for the coupled assay, except that first NADPH was allowed to be produced and then individual hydrolases were added. In the first step, ATP and glucose were employed to yield glucose-6-P and ADP; only then NADP^+^ was added to yield NADPH. To resolve samples, LiCl step gradient was used (see section “Materials and Methods” for details). Lane 1: mock reaction to visualize migration of ATP, ADP, NADP^+^, and NADH (NADP^+^ co-migrates with NADH under these conditions); Lane 2: mock reaction with only ATP and NADP^+^; Lane 3: control reaction for the first step (all ATP is converted to ADP); Lane 4: control reaction for the second step (all NADP^+^ is converted to NADPH); Lane 5–7, MESH-1, Mesh1, and SAH_Mex_ were added.

In order to resolve this question, we tested NADPH hydrolysis under our coupled assay conditions. First, we used ATP (0.5 mM), glucose (1.1 mM), and HK to produce glucose-6-phosphate, which was then used by G6PD to produce NADPH from NADH (supplied at 0.5 mM). The reactions were carried out at 37°C, each for 20 min. Upon the second reaction’s completion, Mesh1, MESH1 or SAH_Mex_ enzymes were added and the reaction was incubated for another 30 min. In this case, 200 ng of each enzyme were added to a 50 μl reaction, which gives an over 11-fold enzyme excess in respect to concentrations used for our standard coupled assay conditions (0.2 μM vs. 18 nM). TLC was used to visualize nucleotides and their derivatives. As demonstrated in Figure 6, we did not detect any NADPH hydrolysis, neither by the human MESH1 enzyme, nor *D. melanogaster* Mesh1 and SAH_Mex_. These results are consistent with observations made by Zhu and Dai (2019) who did not note a disruption in NADP(H)/NAD(H) pools in *E. coli* cells upon Mesh1 overproduction.

Since the TLC assay might be less sensitive than the malachite green assay to detected free phosphate used by Ding et al. (2020), we employed this type of assay under our kinetics assay conditions as well (Table 2). We found no detectable phosphate release when using the same protein concentrations as in the coupled kinetics assay (18 nM) for Mesh1 (*D. melanogaster*) and SAH_Mex_. We observed only negligible phosphate release for MESH1 (human) at the 30 min time point. The procedure was set up to synthesize 300 μM NADPH, followed by addition of either hydrolase. We estimate the detection limit to be at 5 μM phosphate (1.66%). However, when increasing the tested protein concentrations to those that were used in the NADPH-hydrolysis TLC assay (0.2 μM), we did detect phosphate release for MESH1 at other time points (seemingly corresponding to 6.5, 12.1, and 33.3% for the 5, 10, and 30 min, respectively; Table 2) and Mesh1 (6.3 and 14.8% for the 10 and 30 min time points, respectively; Table 2). SAH_Mex_ did not lead to phosphate release in the presence of NADPH under any tested protein concentration.

**TABLE 2:** Release of free phosphate assessed by the malachite green assay and expressed as percentage of NADPH present in the coupled enzymatic assay.

**Enzyme**	**Concentration**	**Phosphate released (%)**		
		**5 min**	**10 min**	**30 min**
Mesh1	18 nM	*no activity*	*no activity*	*no activity*
	0.2 μM	*no activity*	6.3 ± 1.2	14.8 ± 2.2
MESH1	18 nM	*no activity*	*no activity*	1.66 ± 1.4
	0.2 μM	6.5 ± 0.24	12.1 ± 0.19	33.3 ± 2
SAH_Mex_	18 nM	*no activity*	*no activity*	*no activity*
	0.2 μM	*no activity*	*no activity*	*no activity*

*The reactions were set up so that 300 μM NADPH was synthesized, and then Mesh1, MESH1, or SAH_*Mex*_ were added at two different concentrations (18 nM – the same as used in the coupled enzymatic assays; 0.2 μM – the same as in the TLC NADPH hydrolysis assay presented in Figure 6). Samples were removed at 5, 10, and 30 min after hydrolase addition. All reactions were carried out in triplicate. *No activity* – below detection limit, estimated to be 5 μM (1.66%).*

Still, it should be noted that the reported human MESH1 affinity for NADPH (Km) is 120 ± 10 μM, while calculated catalytic efficiency (kcat/Km) is 14.4 ± 1 × 10^3^ s^–1^M^–1^ (Ding et al., 2020). This activity seems rather low in comparison to the catalytic efficiency we found for (p)ppNpp’s and *D. melanogaster* Mesh1, which is about 10-fold higher (see Table 1). Judging by TLC analysis, both enzymes (Mesh1 and MESH1) display similar activities toward (p)ppNpp’s (Figure 1). In this analysis (p)ppNpp’s seem to be better substrates than NADPH. In the (Ding et al., 2020) report, 50 nM MESH1 was used, but the Mn^2+^ concentration which is crucial for most (p)ppNpp hydrolases was the same in both cases (1 mM). However, Mg^2+^ was also included in our assays at 5 mM. In addition, it cannot be excluded that other buffer components had negatively influenced activity of the tested hydrolases toward NADPH. Still, the 30% NADPH hydrolysis by MESH1 inferred from the malachite green assay should have been also observed with the TLC assay (it is a prominent change) and it wasn’t. Thus, it cannot be said with certainty that the observed free phosphate is really released due to NADPH hydrolysis, or is possibly due to hydrolysis of a different substrate, such as e.g., 6-P-dgi which is also produced in our assay. On the other hand, (Ding et al., 2020) worked with pure NADPH substrate. Clearly, while our enzymatic coupled assay is not affected by possible NADPH hydrolysis as the initial reaction rates were not estimated at time points beyond 5 min, further studies are needed to resolve this issue.

## Concluding Remarks

Historically, an abundance of second messenger global regulators is found among incredibly diverse microorganisms; this has led to a fascinating path of constantly increasing complexity of compounds and their functions. First came cAMP and regulation of preferential carbon source utilization. Then (p)ppGpp was associated with arrays of global regulatory responses to multiple nutritional and physical sources of physiological stress. This was followed by multiple sources of cyclic and homo- and hetero-dicyclic purine nucleotides within the same cell interacting to perform specific tasks. The (p)ppApp class of possible nucleotide regulators is now making a debut.

Recently, (p)ppApp was found to be produced by a *Pseudomonas aeruginosa* excreted toxin, which is a part of this organism’s T6SS system (Ahmad et al., 2019). There, cellular toxicity of (p)ppApp has been proposed as due to massive overproduction of (p)ppApp that reaches levels that inhibit PurF, an enzyme which catalyzes the first step in purine nucleotide synthesis, and argued to deplete ATP levels, although GTP depletion is also predicted (Ahmad et al., 2019). The metabolic stress caused by depletion of ATP levels and resulting simply from using it up for overly abundant (p)ppApp synthesis might play an important part here. Also, very recently (Jimmy et al., 2020) used a bioinformatics search to identify numerous presumed toxin-antitoxin clusters, where SAS is the toxin that usually produces ppGpp (toxSAS). Some of these proteins, such as *Cellulomonas marina* toxSAS FaRel can also synthesize ppApp. The authors have demonstrated under *in vivo* conditions, that when this protein is overproduced in the wild type *E. coli* background, it is lethal to the cells. This effect was alleviated by overproduction of three antitoxins–*C. marina* ATfaRel SAH, *Salmonella* phage PVP-SE1 SAH and SSU5 SAH (Jimmy et al., 2020). The authors also showed similar effect for human MESH1 (Jimmy et al., 2020), which was a first reported *in vivo* indication that MESH1 presumably hydrolyzes both, ppGpp and ppApp. Our *in vitro* findings that we present in detail here (first mentioned in Sobala et al., 2019), directly confirm this observation for MESH1.

Still, we would like to stress that since low levels of (p)ppApp are found in growing cells of *E. coli*, *B. subtilis*, and *M. extorquens*, and there is evidence of its transcriptional regulatory activities along with structural data pointing to its unique binding site on *E. coli* RNA polymerase, it is evident that (p)ppApp is not necessarily lethal, but instead might take its place among the second messenger regulators (Bruhn-Olszewska et al., 2018; Sobala et al., 2019).

Nevertheless, assigning regulatory roles for (p)ppApp will require determining its sources of synthesis and hydrolysis among RSH, SAS, and SAH enzymes and whether catalysis is nucleobase specific or mixed. The results obtained here with basically only four hydrolases suggest that a high degree of complexity can be anticipated, since each one of them has different specificity toward the four (p)ppNpp’s. It seems likely that a similar high degree of complexity for fundamental synthetase substrate specificities also exists, let alone diverse regulatory considerations governing their effector properties.

Again, questions arise as to accurate assays needed to assess cellular abundance, physiological functions and enzymatic sources of synthesis and degradation of (p)ppNpp’s. The early TLC assay worked out for (p)ppGpp led to a simple one-dimensional PEI cellulose TLC resolution but this turns out to be inadequate 50 years later. A real advantage of HPLC and MS is that they provide vitally important product purity information. However, these assays are time consuming and are not easily accessible to all. This work describes real time optical coupled assays to monitor the ability of purified proteins to hydrolyze pure, synthesized ppGpp, ppApp, pppGpp, or pppApp for estimating kinetic constants of catalysis. Automated data collection using 96 well microtiter plates greatly facilitates accurate estimates which is crucial for unraveling the complex physiological roles of (p)ppNpp’s.

## Data Availability Statement

The original contributions presented in the study are included in the article/Supplementary Material, further inquiries can be directed to the corresponding author/s.

## Author Contributions

KP and MC conceived this study and wrote the main text. KP, MC, and NET designed the experiments. NET and MC developed the method. KP, NET, BB-O, and TJ performed the experiments. KP, NET, BB-O, MS, and TJ purified the proteins used in this study. KP, BB-O, MC, and NET purified the (p)ppNpp. KP, NET, BB-O, MD, and MC analyzed the data. All authors commented on the manuscript and approved its final version.

## Conflict of Interest

The authors declare that the research was conducted in the absence of any commercial or financial relationships that could be construed as a potential conflict of interest.
