# Supplementary material for: Estimates of RelSeq, Mesh1, and SAHMex Hydrolysis of (p)ppGpp and (p)ppApp by Thin Layer Chromatography and NADP/NADH Coupled Assays
Source: Front Microbiol. 2020 Oct 23;11:581271. doi: 10.3389/fmicb.2020.581271 (PMC7644958; doi:10.3389/fmicb.2020.581271)
Supplement: Supplementary file 1 [file Data_Sheet_1.PDF]

Supplementary Material

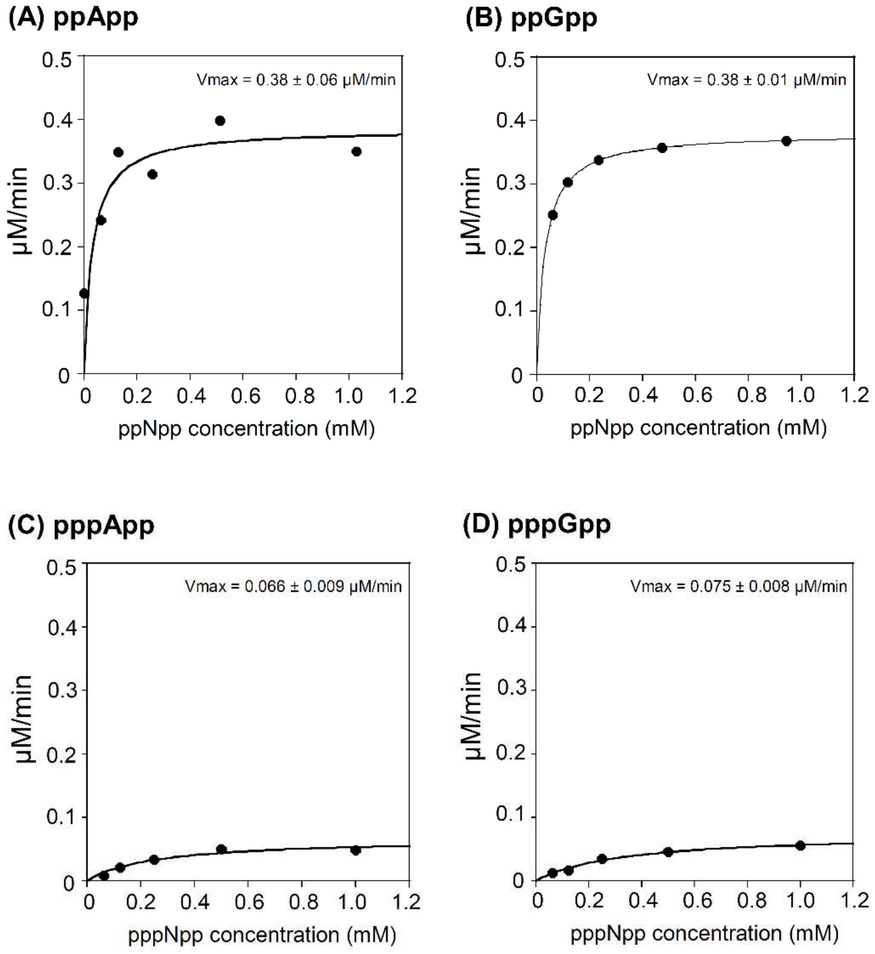

Supplementary Figure 1. Controls: hydrolase independent reaction velocities for (p)ppNpp. (A) For the ppApp assay: ppApp + PEP → pppApp + pyruvate (PK catalyzed reaction). Non-linear regression of initial rates of NADH oxidation yielded an appreciable Vmax required as a correction factor. **(B)** For the ppGpp assay: ppGpp + PEP → pppGpp + pyruvate (PK catalyzed reaction). Non-linear regression of initial rates of NADH oxidation yielded an appreciable Vmax required as a correction factor. **(C)** For the pppApp assay: pppApp + glucose → ppApp +G6P (HK catalyzed reaction). Non-linear regression of initial rates of NADP+ reduction yielded a very weak activity. **(D)** For the pppGpp assay: pppGpp + glucose → ppGpp +G6P (through the NDK or HK catalyzed reaction). Non-linear regression of initial rates of NADP+ reduction yielded a very weak activity.

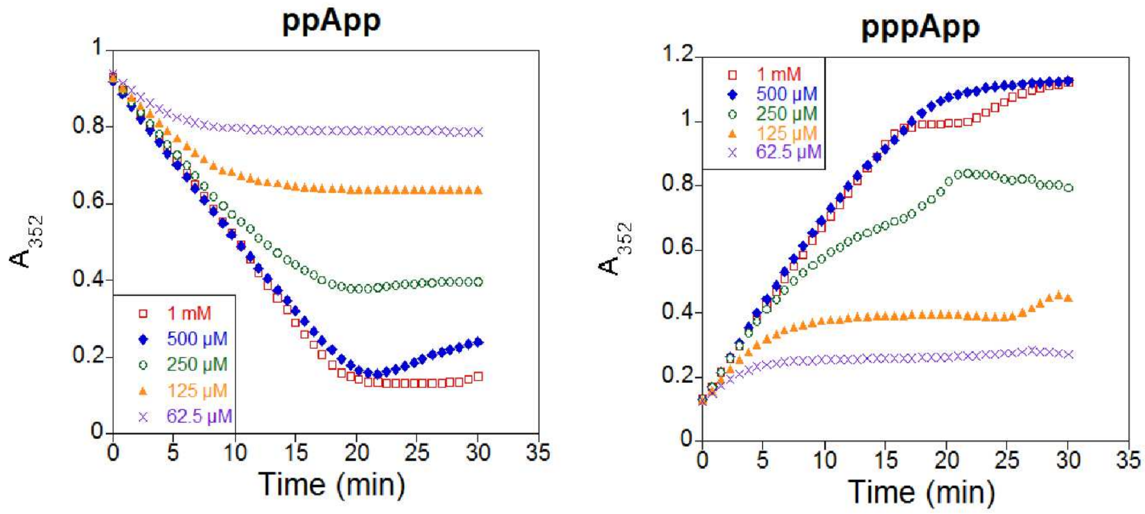

Supplementary Figure 2. Typical raw data depicting NADH oxidation (left) and NADP+ reduction (right) in the coupled enzymatic assay. The data presented was obtained for the enzymatic coupled assays for Mesh1 hydrolysis of ppApp or pppApp as a function of substrate concentration. The duration of initial rates of change used to calculate kinetic constants is appropriately concentration dependent.

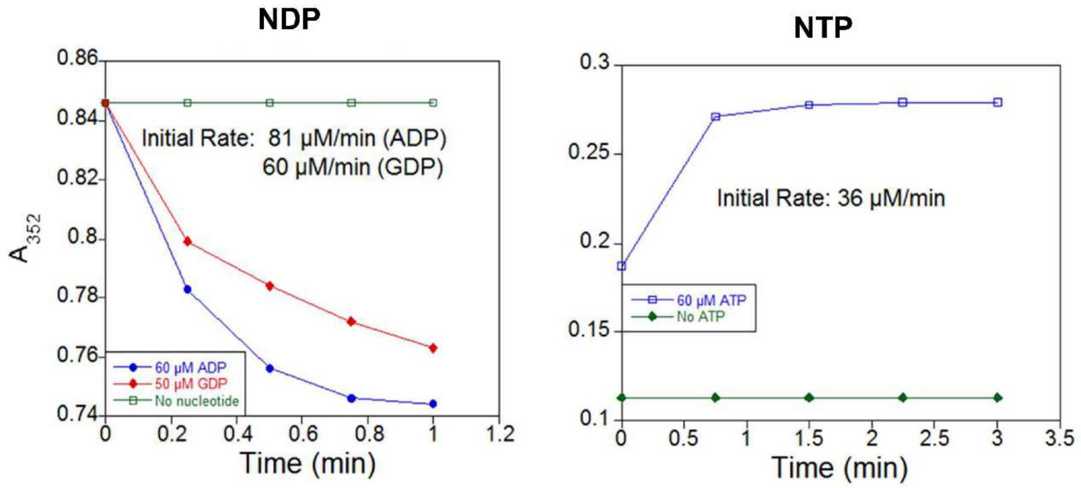

Supplementary Figure 3. Measurements of coupled reaction rates with low substrate concentrations - an indication of the upper limit of (p)ppNpp hydrolysis rates that can be detected when NDP replaces ppNpp or when ATP replaces pppNpp. The values observed exceed rates obtained in experiments with the three tested hydrolases (for ppNpp and pppNpp; see Table 1), indicating upper limits are not exceeded.

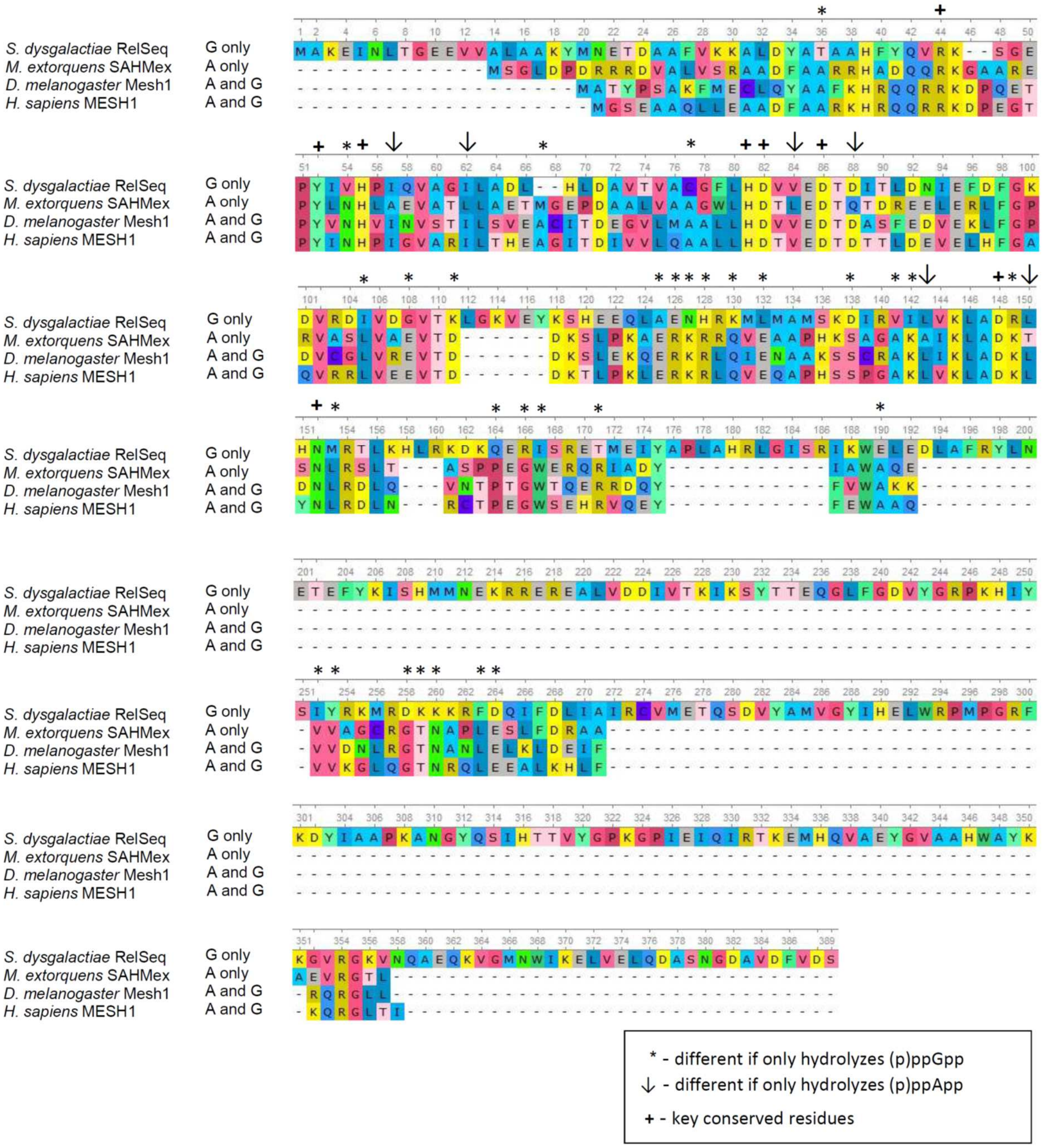

Supplementary Figure 4. Amino acid sequence alignment of Mesh1, MESH1, SAH$_{Mex}$ and RelSeq, done with the UGENE program and the MUSCLE algorithm. RelSeq1-385 fragment was used as the reference. The accession numbers are as follows: *S. dysgalactiae subsp. equisimilis* Rel$_{Seq}$: WP_015016602; *M. extorquens* AM1 SAH$_{Mex}$: ACS40972.1; *D. melanogaster* MESH1: Q9VAM9.1; *H. sapiens* MESH1: Q8N4P3.3. Ability of each enzyme to hydrolyze (p)ppGpp (G) and/or (p)ppApp (A) is indicated. Conserved residues necessary for hydrolysis are based on (Hogg et al., 2004), and are marked with a cross. Residues that are different in RelSeq (unable to hydrolyze (p)ppApp) but the same in the other three enzymes are marked with an asterisk. Residues that are different in SAH$_{Mex}$ (unable to hydrolyze (p)ppGpp) but the same in the other three enzymes are marked with an arrow.
